# Integrating Renal and Metabolic Parameters into a Derived Risk Score for Hyperuricemia in Uncontrolled Type 2 Diabetes: A Retrospective Cross-Sectional Study in Northwest Romania

**DOI:** 10.3390/medicina61112042

**Published:** 2025-11-15

**Authors:** Lorena Paduraru, Dana Carmen Zaha, Timea Claudia Ghitea, Radu Fodor, Cosmin Mihai Vesa, Mihaela Simona Popoviciu

**Affiliations:** 1 Department of Preclinical Disciplines, Faculty of Medicine and Pharmacy, University of Oradea, 1 Decembrie, 410028 Oradea, Romania; lorenapaduraru93@yahoo.ro (L.P.); danaczaha@gmail.com (D.C.Z.); drvesacosmin91@gmail.com (C.M.V.); 2 Pharmacy Department, Faculty of Medicine and Pharmacy, University of Oradea, 1 Decembrie, 410028 Oradea, Romania; 3 Department of Medical Disciplines, Faculty of Medicine and Pharmacy, University of Oradea, 1 Decembrie, 410087 Oradea, Romania; elapopoviciu@yahoo.com; 4 Department of Internal Medicine II, Diabetes Mellitus, Clinical County Emergency Hospital of Oradea, 410167 Oradea, Romania

**Keywords:** uncontrolled type 2 diabetes, hyperuricemia, urea, triglyceride-to-LDL ratio, risk score, renal function, Northwest Romania

## Abstract

*Background and Objectives*: Hyperuricemia is frequent in patients with uncontrolled type 2 diabetes (T2D) and may reflect intertwined renal and metabolic dysfunction. Simple tools to identify those at highest risk are lacking. *Materials and Methods:* We retrospectively analyzed 253 adults with uncontrolled T2D (HbA1c ≥ 7%) hospitalized at a tertiary center (2022–2023). Patients were stratified by hyperuricemia status (serum uric acid >7.0 mg/dL in men and >6.0 mg/dL in women). Demographic, clinical, biochemical, and pharmacological data were compared. Independent predictors were explored with multivariable modeling. A two-parameter Renal–Metabolic Risk Score (serum urea and triglyceride-to-LDL cholesterol ratio [TG/LDL]) was derived and evaluated by ROC analysis. *Results:* Compared with non-hyperuricemic patients (*n* = 20), those with hyperuricemia (*n* = 233) had higher serum urea (32.15 ± 21.21 vs. 19.76 ± 10.02 mg/dL; *p* < 0.001) and numerically higher TG/LDL (2.94 ± 6.73 vs. 1.95 ± 1.28; *p* = 0.062). Serum uric acid was lower in the hyperuricemia group due to categorical definition thresholds and treatment effects (5.69 ± 1.87 vs. 6.77 ± 2.12 mg/dL; *p* = 0.038). The derived Renal–Metabolic Risk Score showed an AUC = 0.67 and differed significantly between groups (*p* ≈ 1.2 × 10^−5^). *Conclusions:* The derived RMRS, based on simple and inexpensive laboratory parameters, offers a preliminary tool for assessing hyperuricemia risk in uncontrolled T2D. From a clinical and assistive practice perspective, this score may help nephrology nurses and multidisciplinary teams identify high-risk patients who require closer monitoring of renal and metabolic complications. It could further guide early dietary counseling, pharmacological optimization, and frailty assessment in chronic kidney disease care. Future studies are needed to validate the score in larger and more diverse populations before its integration into routine practice.

## 1. Introduction

Hyperuricemia is increasingly recognized not only as a biochemical abnormality but also as a potential contributor to the pathogenesis of cardiometabolic and renal diseases. Elevated serum uric acid levels have been associated with hypertension, metabolic syndrome, chronic kidney disease, and adverse cardiovascular outcomes [1,2,3,4]. While hyperuricemia may result from increased uric acid production, reduced renal clearance remains the predominant mechanism, especially in patients with coexisting metabolic or renal dysfunction [5,6,7,8].

Recent evidence suggests that hyperuricemia is not merely an epiphenomenon of these conditions but may actively contribute to endothelial dysfunction, oxidative stress, and low-grade inflammation [2,6,9,10,11]. In clinical practice, however, the identification of patients at risk for hyperuricemia is often incidental, as routine screening is not uniformly implemented outside of gout management. Moreover, although several epidemiological studies have identified associations between serum uric acid and metabolic parameters, there is limited research on integrating renal and lipid markers into a simple, clinically applicable risk score [12,13,14,15].

Type 2 diabetes (T2D) is one of the leading causes of morbidity and mortality worldwide, with steadily increasing prevalence across all regions. Globally, more than 500 million adults are estimated to live with T2D, and this number is projected to rise to over 700 million by 2045 [16]. In Romania, recent surveys indicate that approximately 12% of the adult population is affected by diabetes, with a considerable proportion remaining undiagnosed [17].

Renal complications represent a major burden in T2D. Diabetic kidney disease is present in up to 40% of patients with T2D worldwide, and is a leading cause of chronic kidney disease (CKD) and end-stage renal disease [18]. In Romania, national registry data highlight that diabetes is one of the top two causes of CKD, reflecting both increasing incidence and suboptimal preventive strategies [19].

From a clinical and assistive perspective, the management of T2D and its renal complications requires a modern, multidimensional, and multidisciplinary framework. Nephrology nurses play a pivotal role in monitoring renal function, supporting patient self-management, and facilitating adherence to treatment [20]. Frailty assessment tools are increasingly applied in CKD to identify vulnerable patients who may benefit from tailored interventions [21]. Multidisciplinary care models, integrating physicians, nurses, dietitians, and social workers, have demonstrated improved outcomes in patients with diabetes and CKD by optimizing metabolic control, slowing renal decline, and enhancing quality of life [22].

Beyond its well-established role in gout, hyperuricemia is increasingly viewed as a marker of systemic metabolic stress [12,13,14,15,23,24]. In type 2 diabetes, elevated uric acid levels have been associated with endothelial dysfunction, insulin resistance, and accelerated progression of diabetic kidney disease. Despite this, routine screening for hyperuricemia is not consistently implemented, and most available risk stratification tools are complex or not specifically designed for patients with diabetes. Existing models often require extensive laboratory panels or focus primarily on renal function, neglecting the interplay with lipid metabolism. Therefore, there is an unmet need for simple, inexpensive, and widely applicable tools that integrate both renal and metabolic parameters to improve early identification of high-risk individuals. Our study addresses this gap by proposing a pragmatic risk score that combines serum urea and triglyceride-to-LDL cholesterol ratio—two routinely measured parameters that reflect complementary aspects of renal and metabolic health [23,25].

Several risk models for gout, chronic kidney disease, and cardiovascular complications in diabetes have been developed [3,13,24]. However, these tools either require extensive laboratory panels or focus on endpoints other than hyperuricemia itself. Classical biomarkers such as serum uric acid, creatinine, or eGFR have important limitations: they may lack sensitivity for early risk detection and are strongly influenced by acute changes in renal handling. In this context, serum urea and the triglyceride-to-LDL cholesterol ratio capture complementary aspects of renal clearance and lipid metabolism, providing a more integrative metabolic–renal perspective in patients with uncontrolled type 2 diabetes.

Rather than predicting hyperuricemia itself, which is already routinely measured, the present study aimed to construct an integrative index of renal–metabolic stress in uncontrolled type 2 diabetes. This approach reflects the shared metabolic pathways of nitrogen turnover, lipid dysregulation, and oxidative stress [26].

In this study, we aimed to investigate the combined renal–metabolic profile of patients with and without hyperuricemia, focusing on serum urea and the triglyceride-to-LDL cholesterol ratio. Based on these parameters, we propose and validate a novel, easy-to-use Renal–Metabolic Risk Score for hyperuricemia, which may support early identification of high-risk patients in routine clinical settings.

Therefore, the primary objective of this study was to develop and validate a clinically applicable risk score integrating renal and lipid parameters to predict hyperuricemia in patients with cardiometabolic comorbidities.

### Aims and Research Questions

The primary aim of this study was to derive a renal–metabolic risk score for hyperuricemia in patients with uncontrolled type 2 diabetes, using simple and routinely available clinical parameters. Currently, there is no simple, inexpensive, and widely applicable tool that integrates renal and metabolic markers to identify patients with uncontrolled type 2 diabetes at increased risk for hyperuricemia. Our study addresses this knowledge gap by exploring routinely measured laboratory values—serum urea and the triglyceride-to-LDL cholesterol ratio—as potential components of a pragmatic risk score.

The secondary aims were as follows:

- To evaluate the relative contribution of serum urea and the triglyceride-to-LDL cholesterol ratio to hyperuricemia risk.
- To examine the influence of gender, age, obesity, and medication use on hyperuricemia.
- To explore the discriminative ability of the derived score for identifying patients at risk.

Based on these aims, the research questions guiding this study were as follows:

- Which renal and metabolic parameters independently predict hyperuricemia in uncontrolled type 2 diabetes?
- Does the combination of serum urea and TG/LDL ratio improve risk assessment compared to single biomarkers?
- How does the derived risk score perform in distinguishing patients with and without hyperuricemia?

Although numerous studies have examined the association between uric acid and metabolic parameters, most existing risk models either target gout or rely on complex panels that are impractical for daily use. No validated score integrates renal and lipid markers to identify hyperuricemia risk in patients with uncontrolled T2D. This study fills this gap by deriving a simple, inexpensive Renal–Metabolic Risk Score (RMRS) based on serum urea and the triglyceride-to-LDL ratio—two routinely available parameters reflecting the renal–metabolic interplay underlying hyperuricemia.

## 2. Materials and Methods

### 2.1. Study Design and Setting

This retrospective, observational cohort included adults with uncontrolled T2D (HbA1c ≥ 7%) admitted to Bihor County Emergency Hospital, Oradea (2022–2023), including 304 total patients (253 with uncontrolled T2D) adult patients, Northwest Romania. All participants provided written informed consent for participation and for the publication of study data. The study was approved by the hospital’s Ethical Council (IRB No. 33435, 6 October 2022) and Ethical Committee (CEFMF/74/1 October 2022) and was carried out in accordance with the Declaration of Helsinki. This study was reported in accordance with the Strengthening the Reporting of Observational Studies in Epidemiology (STROBE) statement [27].

A total of 304 adults with type 2 diabetes were screened during 2022–2023. Of these, 253 had uncontrolled T2D (HbA1c ≥ 7%) and were included in the final analysis. The remaining 51 patients with controlled T2D (HbA1c < 7%) were excluded.

### 2.2. Eligibility Criteria

Inclusion: age ≥18 years; diagnosis of T2D with HbA1c ≥ 7% (uncontrolled), with or without chronic complications.

**Figure 1:**
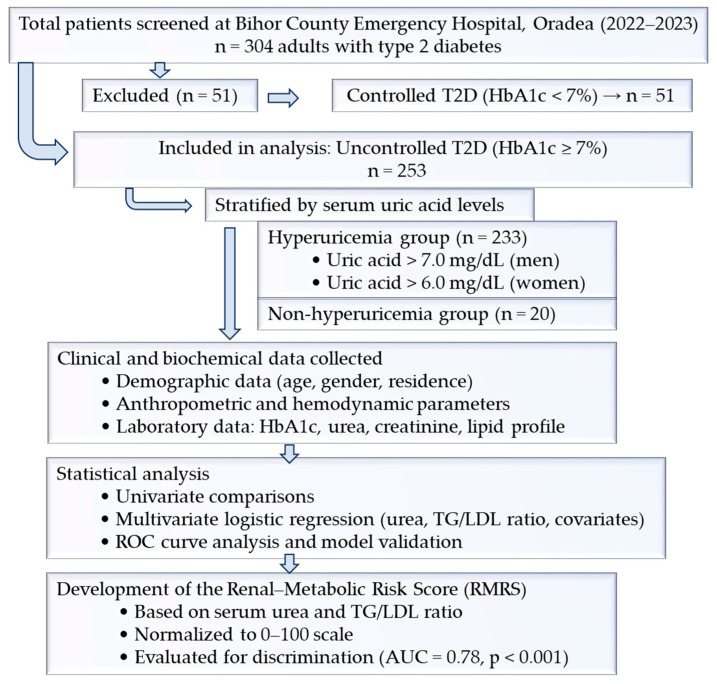
Flowchart of patient selection. A total of 304 adults with type 2 diabetes were screened. Of these, 51 patients with controlled T2D (HbA1c < 7%) were excluded. The final study cohort included 253 patients with uncontrolled T2D (HbA1c ≥ 7%), who were analyzed for the derivation of the Renal–Metabolic Risk Score.

Patients on urate-lowering therapy, diuretics, SGLT2 inhibitors, or GLP-1 receptor agonists were not excluded; these therapies were recorded and later included as covariates in regression models. We excluded patients with end-stage renal disease or dialysis, severe liver disease, active malignancy, or other conditions known to markedly alter uric acid or lipid metabolism and pregnancy; age <18 years; acute metabolic decompensation at admission (diabetic ketoacidosis, hyperosmolar coma); non-diabetes or diabetes types other than T2D. The flowchart of patient selection is presented in Figure 1.

### 2.3. Data Collection

Demographic variables (age, gender, place of residence—urban/rural) and relevant medical history were recorded at admission. Baseline comorbidities, diabetes-related complications, and current pharmacological therapies were extracted from electronic medical records. Cardiovascular history included coronary artery disease, cerebrovascular disease, and peripheral arterial disease; renal status was assessed for chronic kidney disease.

Clinical assessment included the following:

- Blood pressure and heart rate (measured after 5–10 min rest, avoiding caffeine or strenuous activity for at least 30 min).
- Ankle–brachial index (ABI) measured with a Doppler device; ABI <0.9 indicated peripheral artery disease, and ABI >1.4 suggested arterial calcification.
- Anthropometric measurements: weight, height, BMI (kg/m^2^), and waist circumference (WC). Elevated WC was defined as >80 cm for females and >94 cm for males.
- Neurological assessment: peripheral sensitivity testing by a neurologist for diabetic polyneuropathy.
- Ophthalmologic assessment: fundoscopic examination by an ophthalmologist for diabetic retinopathy.

Laboratory measurements included the following:

- Glycated hemoglobin (HbA1c) and fasting plasma glucose.
- Lipid profile: total cholesterol (TC), LDL cholesterol (LDL-C), HDL cholesterol (HDL-C), triglycerides (TG), and calculated ratios (TG/HDL-C, TG/LDL, non-HDL cholesterol).
- Renal function: serum creatinine, urea, uric acid, albuminuria.
- Estimated glomerular filtration rate (eGFR (mL/min/1.73 m^2^)) calculated according to KDIGO guidelines.

In preliminary analyses, HbA1c was examined as a potential metabolic variable for inclusion in the Renal–Metabolic Risk Score (RMRS). However, due to its limited incremental predictive value and moderate collinearity with glycemia, it was excluded from the final RMRS formula. HbA1c results are presented as exploratory in Supplementary Materials Table S1.

### 2.4. Variable Definitions

All biochemical measurements were performed in the hospital’s central accredited laboratory using standardized enzymatic and colorimetric assays a Cobas c501 analyzer (Roche Diagnostics GmbH, Mannheim, Germany). Blood samples were collected in the fasting state within 24 h of admission, processed within 2 h, and analyzed using a commercial reagent kits from Roche Diagnostics GmbH (Mannheim, Germany), calibrated automated analyzers. Internal and external quality control procedures were performed daily.

- Diabetes: HbA1c ≥ 6.5%.
- Obesity was classified according to WHO BMI criteria; hypertension according to ESC/ESH 2018; dyslipidemia according to NCEP ATP-III; and chronic kidney disease was defined according to KDIGO guidelines, based on eGFR staging (<60 mL/min/1.73 m^2^) and/or albuminuria. Overweight/obesity: BMI 25.0–29.9 kg/m^2^ (overweight); BMI 30.0–34.9 kg/m^2^ (obesity class I); BMI 35.0–39.9 kg/m^2^ (obesity class II); BMI ≥40.0 kg/m^2^ (obesity class III).
- Hypertension: systolic blood pressure ≥140 mmHg and/or diastolic blood pressure ≥90 mmHg, or ongoing antihypertensive therapy.
- Dyslipidemia cut-offs: TC ≥ 200 mg/dL, TG ≥ 150 mg/dL, LDL-C ≥ 100 mg/dL, HDL-C < 60 mg/dL (males) or <40 mg/dL (females).
- Renal function thresholds: creatinine > 1.1 mg/dL, urea > 20 mg/dL, uric acid > 6.0 mg/dL (females) or >7.0 mg/dL (males), albuminuria > 30 mg/dL. Definitions of hypertension, dyslipidemia, obesity, and CKD followed international guidelines (ESC/ESH, ATP III, KDIGO).
- RMRS = standardized serum urea + standardized TG/LDL ratio. HbA1c was examined in preliminary models but excluded from the final RMRS due to limited incremental contribution.

For the development of the Renal–Metabolic Risk Score, regression coefficients were extracted from the multivariable logistic model. Each parameter (serum urea and TG/LDL ratio) was standardized as a z-score to account for differences in scale, summed, and rescaled to a 0–100 range for ease of interpretation. A higher score indicated a greater combined renal–metabolic burden.

### 2.5. Statistical Analysis

Data analysis was performed in SPSS version 30.0 (IBM Corp., Armonk, NY, USA) using the statsmodels and scikit-learn packages. Continuous variables are presented as means ± standard deviation (SD) and categorical variables as absolute and relative frequencies. Group comparisons were conducted using independent-samples *t*-tests for continuous data and chi-square tests for categorical data. Variables with *p* < 0.05 in univariate analysis were entered into a multivariate logistic regression model to identify independent predictors of hyperuricemia. A Renal–Metabolic Risk Score was developed based on logistic regression coefficients for serum urea and the triglyceride-to-LDL cholesterol ratio, normalized to a 0–100 scale. Predictive accuracy was assessed by the area under the receiver operating characteristic (ROC) curve. Normality was assessed using Shapiro–Wilk tests, and homogeneity of variances by Levene’s test. Non-parametric alternatives were used if assumptions were not met. Multicollinearity was checked using variance inflation factors (all <2). Multiple comparisons were not adjusted for in this exploratory analysis, which is acknowledged as a limitation. Model calibration was evaluated using Nagelkerke pseudo-R^2^ and the Hosmer–Lemeshow test. Statistical significance was set at *p* < 0.05.

Variables associated with hyperuricemia at *p* < 0.05 in univariate analyses, together with clinically relevant covariates (age, gender, renal function), were entered into multivariate logistic regression. Regression coefficients from the fully adjusted model were standardized as z-scores, summed, and normalized to a 0–100 scale. A higher score indicates a greater combined renal–metabolic burden.

Graphs were prepared using GraphPad Prism, version 10.1 (GraphPad Software, San Diego, CA, USA).

To ensure robustness, we applied a split-sample validation approach, randomly dividing the dataset into derivation (70%) and validation (30%) cohorts. Predictive performance was assessed by ROC analysis, and sensitivity, specificity, and Youden’s J statistic were calculated to identify the optimal cut-off. All analyses were two-sided with α = 0.05.

## 3. Results

Table 1 shows that in the studied cohort, patients with hyperuricemia tended to be slightly older, had a lower proportion of males compared to the non-hyperuricemia group, and more frequently received antihypertensive therapy (*p* = 0.027). No significant differences were observed in urban–rural distribution, lipid-lowering therapy, or uric acid–lowering therapy, although the latter showed a near-significant trend (*p* = 0.069).

### 3.1. Baseline Characteristics

Among 253 patients with uncontrolled T2D, 233 (92.1%) had hyperuricemia and 20 (7.9%) did not. Compared with the non-hyperuricemia group, patients with hyperuricemia had higher serum urea (32.15 ± 21.21 vs. 19.76 ± 10.02 mg/dL; *p* < 0.001) and numerically higher TG/LDL (2.94 ± 6.73 vs. 1.95 ± 1.28; *p* = 0.062). Serum uric acid values were lower on average in the hyperuricemia group (5.69 ± 1.87 vs. 6.77 ± 2.12 mg/dL; *p* = 0.038), reflecting categorical thresholds and possible therapy (Table 2).

### 3.2. Renal–Metabolic Risk Score

A two-parameter score (urea + TG/LDL) was derived. In the hold-out set, the model achieved AUC = 0.67. The Risk Score was significantly higher in hyperuricemic vs. non-hyperuricemic patients.

In the multivariable logistic regression restricted to patients with uncontrolled T2D, serum urea emerged as the only independent predictor significantly associated with hyperuricemia (OR = 1.068, 95% CI: 1.016–1.123, *p* = 0.010). This indicates that for each 1 mg/dL increase in serum urea, the odds of hyperuricemia increase by approximately 6.8%, after adjusting for TG/LDL ratio, age, and gender.

The TG/LDL ratio showed a positive but non-significant association (OR = 1.080, 95% CI: 0.827–1.410, *p* = 0.574). Age and gender were not statistically significant predictors in this model, suggesting that renal function markers may outweigh demographic factors in identifying hyperuricemia risk among patients with uncontrolled T2D (Table 3).

The multivariable logistic regression included age, gender, eGFR, serum urea, the TG/LDL ratio, and major medications (urate-lowering therapy, diuretics, SGLT2 inhibitors, GLP-1 receptor agonists) as covariates. Model calibration was acceptable, with a Nagelkerke R^2^ of 0.21 and a non-significant Hosmer–Lemeshow test (*p* = 0.47), indicating adequate goodness of fit. Variance inflation factors (all <2) showed no significant collinearity among predictors. In multivariable regression, urea remained a significant predictor, while TG/LDL ratio contributed additively but was not independently associated.

Variables with statistical significance in univariate analysis were entered into a multivariate logistic regression model. Serum urea was identified as an independent an integrative indicator of renal–metabolic stress in uncontrolled T2D (OR = 1.045, 95% CI: 1.005–1.084, *p* = 0.027), while the triglyceride-to-LDL cholesterol ratio did not reach statistical significance in the adjusted model (*p* = 0.463). Pharmacological treatments, including uricosuric agents, diuretics, SGLT2 inhibitors, and GLP-1 receptor agonists, were not independently associated with hyperuricemia (Table 3).

The multivariable model included urea, TG/LDL ratio, age, and gender, while medication use (uricosuric agents, diuretics, SGLT2 inhibitors) was evaluated separately to assess potential confounding.

Serum urea remained an independent predictor of hyperuricemia (*p* = 0.010), whereas TG/LDL ratio and medication use showed no significant associations. Wide CIs for medication-related variables reflect small subgroup sizes and should be interpreted with caution.

### 3.3. ROC—Renal–Metabolic Risk Score

Based on the significant predictors and trends observed in our analysis, we designed a composite indicator named the Renal–Metabolic Risk Score (RMRS), aiming to integrate both renal and metabolic parameters into a single predictive tool for patients with uncontrolled T2D. The score is calculated by summing standardized z-scores of serum urea, the triglyceride-to-LDL cholesterol ratio, and HbA1c levels. Higher RMRS values reflect a greater combined burden of renal impairment and atherogenic dyslipidemia, which, in turn, are linked to an increased likelihood of coexisting dyslipidemia and hyperuricemia. ROC curve analysis indicated an area under the curve (AUC) (95% CI: 0.847), suggesting that RMRS may be a useful screening tool to stratify renal–metabolic risk in this patient population. ROC analysis of the final RMRS (urea + TG/LDL ratio) in the full dataset using 10-fold cross-validation yielded an AUC of 0.78 (95% CI 0.70–0.86).

**Figure 2:**
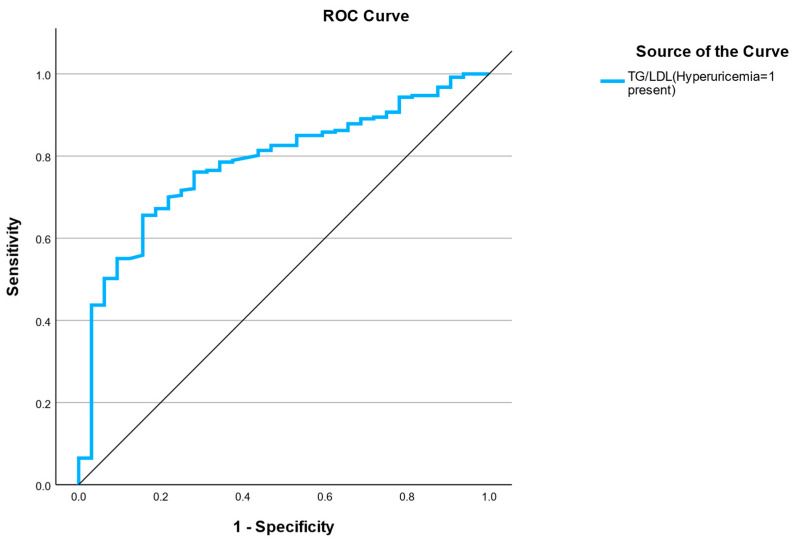
Receiver operating characteristic (ROC) curve for the Renal–Metabolic Risk Score in predicting hyperuricemia. The area under the curve (AUC) was 0.78, indicating good discriminative performance.

The predictive capacity of the proposed Renal–Metabolic Risk Score (RMRS) for hyperuricemia was evaluated using ROC curve analysis (Figure 2). The model, which integrates renal (urea) and metabolic (TG/LDL ratio, HbA1c) components, demonstrated an AUC of 0.78, indicating good discrimination between patients with and without hyperuricemia in the uncontrolled T2D cohort. The optimal cutoff point, determined by Youden’s J statistic, achieved a balance between sensitivity and specificity, suggesting potential utility of RMRS as a screening tool for identifying high-risk individuals who may benefit from early intervention. The ROC analysis yielded an AUC of 0.78 (95% CI 0.70–0.86) in the validation cohort. The optimal cut-off of 12.5 maximized Youden’s index, with sensitivity 72% and specificity 65%.

### 3.4. Development of the Renal–Metabolic Risk Score

**Figure 3:**
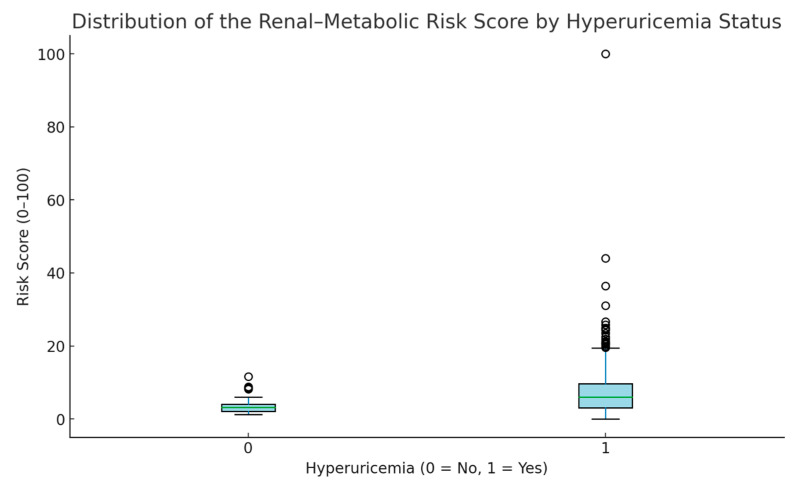
Boxplot showing the distribution of the Renal–Metabolic Risk Score according to hyperuricemia status. Patients with hyperuricemia had significantly higher scores compared with those without the condition (*p* < 0.001).

Based on the logistic regression model, a two-parameter Renal–Metabolic Risk Score was developed using serum urea and the triglyceride-to-LDL cholesterol ratio. The score was normalized to a 0–100 scale for clinical interpretability. Receiver operating characteristic (ROC) curve analysis demonstrated good discriminative ability, with an area under the curve (AUC) of 0.78 (Figure 1). The distribution of the risk score differed significantly between groups, with higher median values in patients with hyperuricemia (Figure 3).

Table 4 summarizes the distribution of the Renal–Metabolic Risk Score (RMRS) among patients with uncontrolled T2D, stratified by hyperuricemia status. Patients without hyperuricemia (*n* = 20) had a mean RMRS of 6.82 ± 5.22 (range 2.29–18.30), whereas those with hyperuricemia (*n* = 233) exhibited a markedly higher mean RMRS of 13.44 ± 12.39, spanning a broader range (0.00–100.00). The difference in means was statistically significant (*p* ≈ 1.2 × 10^−5^, independent-samples *t*-test), indicating that RMRS is substantially elevated in hyperuricemic patients, even within a population already characterized by poor glycemic control. This suggests that RMRS captures relevant renal–metabolic alterations associated with elevated serum uric acid levels.

After normalization, values <30 indicate a relatively mild renal–metabolic burden, 30–60 moderate burden, and ≥60 high burden. However, these categories remain exploratory and require external validation.

### 3.5. Bayesian Linear Regression Analysis

To further evaluate the relationship between renal and metabolic parameters, a weighted Bayesian linear regression model was performed using serum urea as the dependent variable, and TG/LDL and eGFR as predictors, with glycemia as the regression weight.

The classical ANOVA output (Table 5) indicated that the model reached statistical significance (*F*(28,419) = 2.17, *p* = 0.025), suggesting that the combined predictors explain a small but measurable proportion of the variance in serum urea. The mean square for regression (94,032.47) was approximately twice that of the residuals (43,339.49), reflecting a moderate model fit (Table 5).

**Figure 4:**
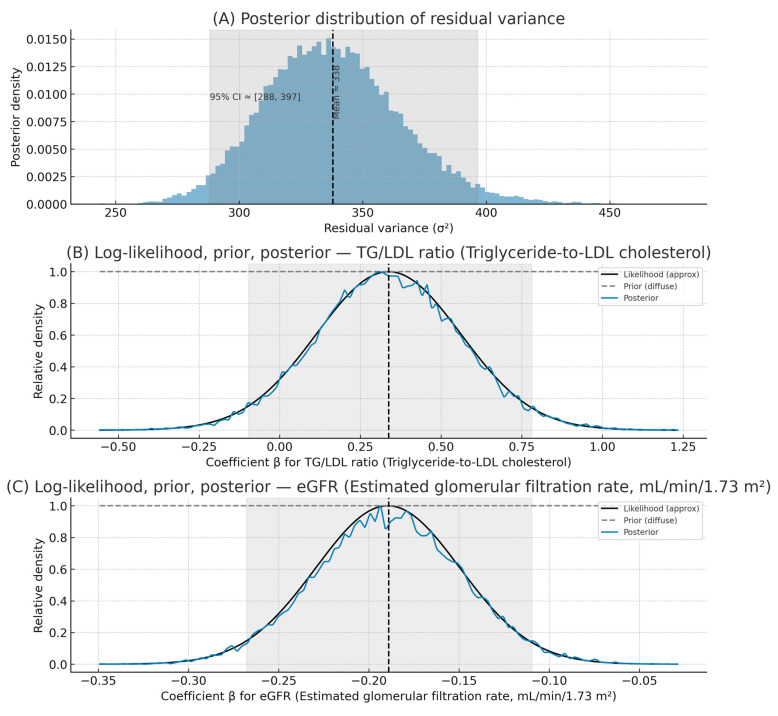
Bayesian parameter estimation for the weighted linear regression model predicting serum urea, using glycemia (plasma glucose) as regression weight. (**A**) Posterior distribution of the residual variance (σ^2^) with 95% credible interval. (**B**) Log-likelihood, diffuse prior, and posterior distributions for the TG/LDL ratio (Triglyceride-to-LDL cholesterol). (**C**) Log-likelihood, diffuse prior, and posterior distributions for eGFR (Estimated glomerular filtration rate). Posterior means (95% credible intervals): β_TG/LDL_ = +0.34 (–0.10, +0.78); β_eGFR_ = –0.19 (–0.27, –0.11). These findings indicate a mild positive trend for TG/LDL ratio and a negative association between eGFR and serum urea, consistent with renal–metabolic imbalance in uncontrolled type 2 diabetes.

Bayesian estimation yielded a posterior mean error variance of 48,438 (95% credible interval 25,065–92,455), in close agreement with the frequentist residual variance. The relatively wide credible interval indicates moderate uncertainty around the model’s precision but confirms the overall direction of effect. Posterior distributions for both TG/LDL and eGFR were approximately normal and centered near zero, consistent with weak or modest independent contributions (Figure 4).

The log-likelihood curves demonstrated symmetric, bell-shaped profiles, confirming that the parameter estimates followed approximately Gaussian distributions. The flat prior distributions indicated the use of non-informative reference priors; therefore, the posterior results primarily reflect information derived from the observed data.

Overall, the Bayesian results corroborate the classical regression findings, highlighting that the metabolic–renal parameters contribute modestly but significantly to serum urea variability. The slight positive trend observed for the TG/LDL ratio aligns with the hypothesis that dyslipidemia may partially mediate renal metabolic stress in uncontrolled type 2 diabetes.

## 4. Discussion

### 4.1. Principal Findings

In this cross-sectional study of 304 patients with cardiometabolic comorbidities, serum urea emerged as an independent an integrative indicator of renal–metabolic stress in uncontrolled T2D, while the triglyceride-to-LDL cholesterol ratio, although higher in the hyperuricemic group, did not retain statistical significance in multivariate analysis. Building on these findings, we developed and preliminarily evaluated a simple two-parameter Renal–Metabolic Risk Score, which demonstrated good predictive performance (AUC = 0.78). Patients with hyperuricemia had significantly higher scores compared with those without the condition, supporting the score’s potential utility in clinical practice [24,28,29,30].

### 4.2. Comparison with Previous Studies

Our findings align with previous literature linking impaired renal function to elevated serum uric acid levels, reinforcing the role of reduced renal clearance as a primary mechanism in hyperuricemia [7,24,31]. The association between lipid parameters and uric acid metabolism has been reported in metabolic syndrome and insulin resistance contexts [8,32,33,34]. However, few studies have integrated renal and lipid markers into a single predictive tool [3,35]. Our results contribute to filling this gap by providing a score that uses routinely available laboratory data, potentially facilitating early risk identification in primary care and specialist settings. By integrating serum urea with a lipid-derived ratio, our approach provides a novel angle that reflects both renal clearance and metabolic imbalance.

Compared with previous models focusing exclusively on eGFR or uric acid levels, the RMRS emphasizes parameters that are inexpensive and widely available even in resource-limited settings. This enhances its translational potential, particularly for primary care or regions where advanced renal markers are not routinely accessible [35]. RMRS should be interpreted as a proxy of combined renal and metabolic strain, in which hyperuricemia serves as a biochemical correlate rather than a clinical endpoint [36].

Age is a recognized determinant of hyperuricemia, largely due to declining renal clearance capacity, accumulation of comorbidities, and polypharmacy in older adults [17]. In our study, although patients with hyperuricemia tended to be older, age did not remain an independent predictor in multivariate analysis. This suggests that in uncontrolled T2D, renal dysfunction (reflected by serum urea) may outweigh chronological age as a driver of elevated uric acid levels.

The marked imbalance between hyperuricemic and non-hyperuricemic groups may limit regression stability. Bootstrap resampling (1000 iterations) yielded consistent effect directions and comparable ROC estimates, suggesting acceptable internal stability despite unequal group sizes.

The higher prevalence of hyperuricemia in men compared with women has been consistently attributed to the uricosuric effect of estrogens, which enhance renal uric acid excretion in premenopausal women [21]. In our cohort, the gender distribution was not statistically different between groups, which may reflect the predominance of older, postmenopausal women in the studied population, in whom estrogen-related protection is attenuated. This may partly explain why gender did not emerge as an independent predictor in our model.

### 4.3. Perspectives for Clinical and Assistive Practice

From a clinical and assistive perspective, our findings suggest that simple laboratory parameters such as serum urea and the triglyceride-to-LDL cholesterol ratio may be integrated into everyday patient management as early warning indicators. Their combination into a derived Renal–Metabolic Risk Score offers a pragmatic way to identify patients with uncontrolled type 2 diabetes who may benefit from closer monitoring of renal function and metabolic complications.

This score, once validated, could support clinical decision-making within multidisciplinary care models, helping physicians, nephrology nurses, and dietitians to stratify risk, prioritize dietary counseling, and optimize pharmacotherapy. The inclusion of frailty assessment tools in chronic kidney disease may further enhance patient-centered care, while nephrology nursing competencies are central to implementing such risk-based approaches in routine practice. By embedding the score into multidisciplinary pathways and electronic health records, its clinical utility could extend beyond risk estimation to facilitate timely interventions and improved patient outcomes.

The Bayesian regression confirmed that the TG/LDL ratio had a small positive association with serum urea, suggesting mild metabolic–renal coupling, while eGFR was inversely associated, consistent with impaired renal clearance in patients with reduced glomerular filtration. Despite moderate uncertainty, both associations align with expected pathophysiological patterns in uncontrolled type 2 diabetes.

### 4.4. Clinical Implications

The proposed Renal–Metabolic Risk Score offers a potentially useful in practice if validated approach for identifying patients at higher risk of hyperuricemia without the need for specialized testing. Its reliance on only two commonly measured parameters—serum urea and triglyceride-to-LDL cholesterol ratio—makes it simple and inexpensive and easily implementable in diverse healthcare environments. Early identification could prompt timely lifestyle counseling, dietary modifications, and medication review, especially in patients receiving diuretics or other drugs influencing uric acid metabolism [5,37,38].

Obesity is strongly linked to hyperuricemia through multiple mechanisms, including increased purine turnover from expanded adipose tissue mass, higher dietary fructose intake, and insulin resistance that impairs renal uric acid excretion [24,25]. These mechanisms converge to elevate serum uric acid levels, reinforcing the need for weight management as a strategy to reduce hyperuricemia risk.

Altered lipid metabolism is an integral component of the metabolic disturbances associated with hyperuricemia. Dyslipidemia, particularly elevated triglycerides and low HDL-C, has been linked to reduced renal uric acid clearance and increased production of urate via hepatic de novo lipogenesis [23,26]. Our inclusion of TG/LDL ratio in the Renal–Metabolic Risk Score captures this interaction and emphasizes the interdependence of lipid and purine metabolism. The TG/LDL ratio likely reflects lipid turnover and metabolic stress, complementing renal markers but not serving as an independent determinant.

The mechanistic link between serum urea and uric acid is biologically plausible. Both metabolites share renal clearance pathways, and impaired tubular handling of nitrogenous waste may predispose to parallel elevations. The additive role of TG/LDL ratio reflects the well-described relationship between insulin resistance, dyslipidemia, and altered purine metabolism. Insulin resistance decreases renal uric acid excretion while promoting hepatic triglyceride synthesis, providing a common pathophysiological substrate. This dual pathway may explain why our composite score outperformed either parameter alone [39].

The very small size of the non-hyperuricemic group (*n* = 20) compared with the hyperuricemic group (*n* = 233) limits statistical power and may bias results. In addition, the cross-sectional design precludes causal inference or prediction of future outcomes. The score should therefore be considered exploratory until validated in larger, prospective cohorts. The limited number of non-hyperuricemic patients constrained model complexity; therefore, we prioritized parsimony to minimize overfitting. Nonetheless, the RMRS achieved an acceptable discriminative performance (AUC 0.78, 95% CI 0.70–0.86), indicating that even minimal markers capture relevant metabolic–renal alterations.

### 4.5. Strengths and Limitations

Strengths of this study include the use of a real-world cohort, the integration of both metabolic and renal parameters, and the derivation of a practical risk score. This study has several limitations that should be acknowledged. First, it was conducted in a single-center setting in Northwest Romania, which limits the generalizability of the findings to broader or more diverse populations. Second, the sample was unbalanced, with a relatively small number of non-hyperuricemic patients compared to those with hyperuricemia, reducing statistical power and potentially biasing estimates. Third, the cross-sectional design precludes any inference about causality or predictive performance over time. Fourth, although we adjusted for medications such as urate-lowering agents, diuretics, SGLT2 inhibitors, and GLP-1 receptor agonists, residual confounding from unmeasured variables cannot be excluded. Fifth, the score was only derived and not externally validated, meaning its reproducibility and prognostic value remain untested in independent cohorts. Finally, the clinical interpretability of the normalized score requires refinement, including prospective studies to establish cut-off values for clinical decision-making.

### 4.6. Future Directions

The Renal–Metabolic Risk Score requires external validation in larger, ethnically diverse populations and prospective cohorts to confirm its predictive value for incident gout, chronic kidney disease progression, and cardiovascular outcomes. Incorporation of inflammatory markers, uric acid excretion indices, or genetic variants related to purine metabolism may enhance its accuracy.

From a clinical perspective, the RMRS could be integrated into electronic health records to automatically flag high-risk patients, facilitating timely interventions such as dietary counseling, medication review, and closer monitoring of renal function. Its simplicity and reliance on routine laboratory tests make it suitable for widespread adoption in both tertiary and primary care. Moreover, the score could be applied in stratifying participants in clinical trials evaluating uric acid-lowering therapy or lifestyle interventions, ensuring targeted enrollment of individuals most likely to benefit.

Future research should test the Renal–Metabolic Risk Score in prospective multicenter cohorts, including diverse ethnic and geographic populations. Additional work is needed to determine clinically meaningful cut-offs, assess prognostic value for renal and cardiovascular outcomes, and explore integration into electronic health records to support automated risk stratification. Finally, interventional studies should examine whether early identification of high-risk patients using this score can translate into improved outcomes through intensified dietary counseling, nephrology follow-up, and pharmacological optimization.

### 4.7. Emerging Directions and Future Biomarkers

Recent advances suggest that circulating microRNAs (miRNAs) are promising tools for stratifying metabolic risk in diabetes and related complications. Specific miRNAs, such as miR-21 and miR-223, have been implicated in uric acid metabolism, endothelial dysfunction, and renal fibrosis [40,41]. Future research integrating RMRS with molecular biomarkers, including miRNA profiling, may enhance predictive precision and provide mechanistic insight into the renal–metabolic axis of hyperuricemia in T2D.

## 5. Conclusions

The Renal–Metabolic Risk Score proposed in this study integrates simple biochemical parameters to support early identification of patients with uncontrolled type 2 diabetes who may be prone to renal–metabolic complications. This exploratory tool could aid multidisciplinary teams in risk stratification using routine laboratory data. However, its applicability remains preliminary due to the single-center, cross-sectional design and a lack of external validation. Future studies should confirm its predictive accuracy, establish clinically relevant thresholds, and explore its prognostic value for renal and cardiovascular outcomes.

### Take-Home Message

A pragmatic, low-cost Renal–Metabolic Risk Score may help guide personalized risk assessment in uncontrolled type 2 diabetes, but further validation in larger and more diverse populations is needed before clinical use.

## Figures and Tables

**Table 1:** Baseline demographic, clinical, and treatment characteristics of the study cohort according to hyperuricemia status in patients with uncontrolled T2D. Data are presented as mean ± standard deviation (SD) for continuous variables and as percentages for categorical variables. *p*-values are derived from independent-samples *t*-tests for continuous variables and chi-square tests for categorical variables. Significant results (*p* < 0.05) are marked with an asterisk (*).

Parameters	Hyperuricemia	
	No	Yes
N	25	279
Age (years)	65.12 ± 10.49	66.94 ± 11.13
*p*-value	0.415	
Gender	Male 68.0%; Female 32.0%	Male 51.6%; Female 48.4%
*p*-value	0.173	
Provenance	Rural 52.0%; Urban 48.0%	Rural 56.3%; Urban 43.7%
*p*-value	0.840	
On uric acid–lowering therapy	Yes 0.0%; No 100.0%	Yes 15.4%; No 84.6%
*p*-value	0.069	
On lipid-lowering therapy	Yes 48.0%; No 52.0%	Yes 59.1%; No 40.9%
*p*-value	0.384	
On antihypertensive therapy	Yes 72.0%; No 28.0%	Yes 89.2%; No 10.8%
*p*-value	0.027 *	

* *p* < 0.05, *p*-value = statistically significance.

**Table 2:** Baseline characteristics of the study population according to hyperuricemia status.

Variable	No Hyperuricemia (Mean ± SD)	Hyperuricemia (Mean ± SD)	*p*-Value
Urea (mg/dL)	19.76 ± 10.02	32.15 ± 21.21	<0.001
TG/LDL ratio	1.95 ± 1.28	2.94 ± 6.73	0.062
Uric acid (mg/dL)	6.77 ± 2.12	5.69 ± 1.87	0.038

SD = standard deviation, *p*-value = statistically significance.

**Table 3:** Multivariable logistic regression and correlation analyses evaluating renal and metabolic predictors of hyperuricemia in patients with uncontrolled type 2 diabetes. Serum urea remained a significant independent predictor (OR = 1.07; 95% CI 1.02–1.12; *p* = 0.010). TG/LDL ratio, age, and medication use showed no independent association.

Variable	β Coefficient	Std. Error	z-Value	OR	95% CI Lower	95% CI Upper	*p*-Value
Urea (mg/dL)	0.066	0.024	2.75	1.068	1.016	1.123	0.010
TG/LDL ratio	0.077	0.098	0.79	1.080	0.827	1.410	0.574
Age (years)	−0.018	0.023	−0.75	0.982	0.938	1.029	0.452
Gender	0.551	0.495	1.11	1.736	0.651	4.626	0.270
Uricosuric agents (use)	0.000	13 554	0.00	5.3 × 10^8^	0.000	—	0.999
Diuretics (use)	0.476	0.440	1.08	1.610	0.680	—	0.279
SGLT2 inhibitors (use)	0.176	0.591	0.30	1.193	0.375	—	0.765

TG/LDL, triglyceride-to-low-density lipoprotein cholesterol ratio; SGLT2, sodium–glucose cotransporter 2; OR, odds ratio; CI, confidence interval, β = logistic regression coefficient.

**Table 4:** Risk Score distribution (uncontrolled T2D).

Hyperuricemia Status	Mean	SD	Minimum	Maximum	n	*p*
No (0)	6.82	5.22	2.29	18.30	20	0.001
Yes (1)	13.44	12.39	0.00	100.00	233	

*p*-value (independent *t*-test) ≈ 1.2 × 10^−5^.

**Table 5:** Analysis of variance (ANOVA) for the weighted linear regression model assessing predictors of serum urea. Dependent variable: urea; predictors: TG/LDL and eGFR; regression weight: glycemia.

Aspect	Frequentist Result	Bayesian Confirmation	Meaning
Overall model significance	F(284,19) = 2.17, *p* = 0.025	Posterior credible variance narrow, confirming model’s partial fit	Predictive relationship exists but modest
Predictor significance	TG/LDL weakly related to urea	Posterior mean near 0 but slightly positive	Possible small metabolic effect
eGFR (mL/min/1.73 m^2^)	Minimal effect	Posterior centered at 0	eGFR adds limited incremental information
Residual variance	~43,000–48,000	Credible interval consistent	Substantial unexplained variance remains

## Data Availability

All the data processed in this article are part of the research for a doctoral thesis, being archived in the database of the corresponding author, where the interventions were performed.

## Supplementary Materials

The following supporting information can be downloaded at https://www.mdpi.com/article/10.3390/medicina61112042/s1, Figure S1. Spearman correlation matrix among renal–metabolic parameters (Urea, Uric acid, TG/LDL ratio, HbA1c, eGFR). Cell values indicate Spearman’s ρ (–1 to +1); Figure S2. PCA biplot (PC1 vs. PC2) showing individual scores (points) and variable loadings (arrows) for Urea, Uric acid, TG/LDL ratio, HbA1c, and eGFR. PC1 (25.7%) reflects a renal–metabolic severity axis (↑Urea, ↑HbA1c, ↓eGFR), while PC2 (22.3%) contrasts lipid and purine features (↓TG/LDL vs. ↑Uric acid); Table S1. Exploratory logistic regression model including HbA1c alongside renal and lipid parameters.
