# Supplementary material for: Integrating Renal and Metabolic Parameters into a Derived Risk Score for Hyperuricemia in Uncontrolled Type 2 Diabetes: A Retrospective Cross-Sectional Study in Northwest Romania
Source: Medicina (Kaunas). 2025 Nov 15;61(11):2042. doi: 10.3390/medicina61112042 (PMC12654377; doi:10.3390/medicina61112042)
Supplement: Supplementary file 1 [file medicina-61-02042-s001.zip › medicina-3953182-supplementary.pdf]

Supplementary file

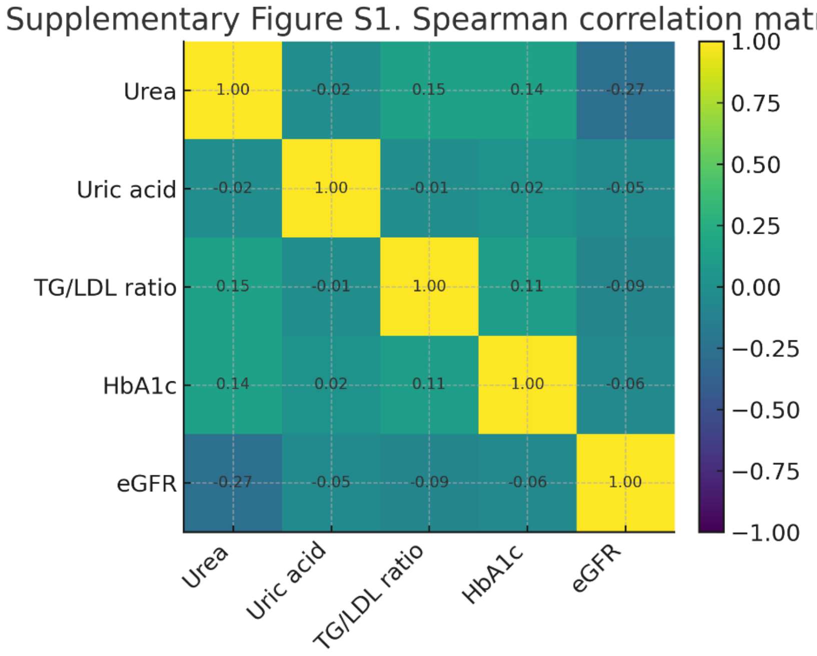

Supplementary Figure S1. Spearman correlation matrix among renal–metabolic parameters (Urea, Uric acid, TG/LDL ratio, HbA1c, eGFR). Cell values indicate Spearman's ϱ (–1 to +1).

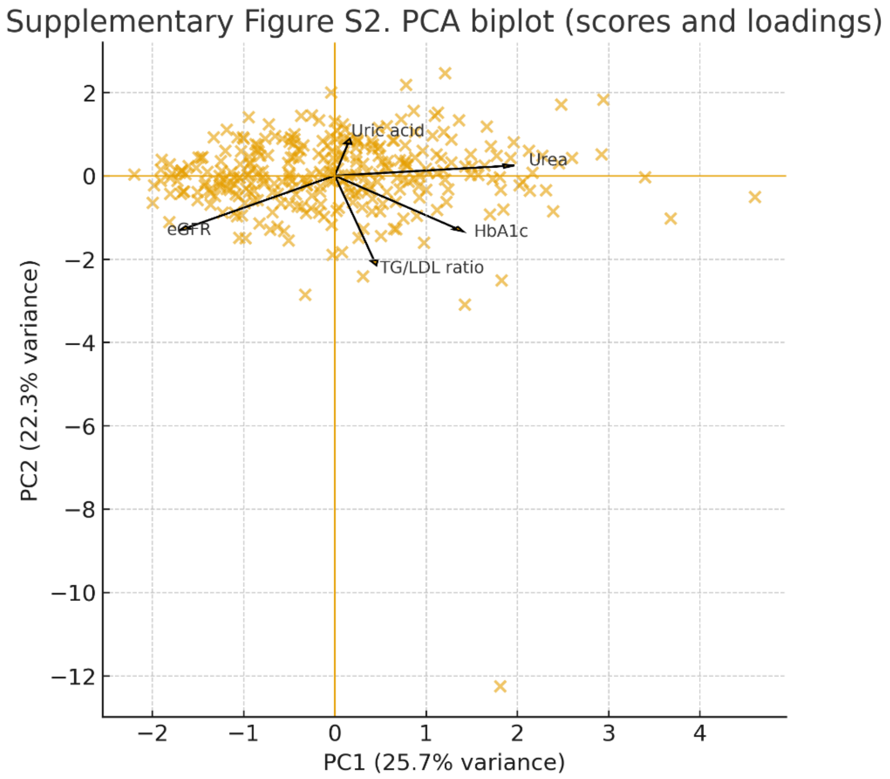

Supplementary Figure S2. PCA biplot (PC1 vs. PC2) showing individual scores (points) and variable loadings (arrows) for Urea, Uric acid, TG/LDL ratio, HbA1c, and eGFR. PC1 (25.7%) reflects a renal–metabolic severity axis (↑Urea, ↑HbA1c, ↓eGFR), while PC2 (22.3%) contrasts lipid and purine features (↓TG/LDL vs. ↑Uric acid).

Supplementary Table S1. Exploratory logistic regression model including HbA1c alongside renal and lipid parameters.

Variable	β Coefficient	Std. Error	z-value	OR	95 % CI Lower	95 % CI Upper	p-value
Urea (mg/dL)	0.060	0.025	2.37	1.062	1.010	1.118	0.018
TG/LDL ratio	0.070	0.099	0.71	1.073	0.821	1.403	0.478
HbA1c (%)	0.118	0.152	0.78	1.125	0.840	1.507	0.437

The inclusion of HbA1c did not improve model discrimination (AUC = 0.77 vs. 0.78 without HbA1c), confirming its exploratory role and exclusion from the final RMRS.
